# Heart Rate Variability-Derived Thresholds for Exercise Intensity Prescription in Endurance Sports: A Systematic Review of Interrelations and Agreement with Different Ventilatory and Blood Lactate Thresholds

**DOI:** 10.1186/s40798-023-00607-2

**Published:** 2023-07-18

**Authors:** Sebastian Kaufmann, Thomas Gronwald, Fabian Herold, Olaf Hoos

**Affiliations:** 1 Center for Sports and Physical Education, Faculty of Human Sciences, Julius-Maximilians-University Wuerzburg, Am Hubland/Sports Center, 97074 Würzburg, Germany; 2 Institute of Interdisciplinary Exercise Science and Sports Medicine, MSH Medical School Hamburg, Hamburg, Germany; 3 Research Group Degenerative and Chronic Diseases, Movement, Faculty of Health Sciences Brandenburg, University of Potsdam, Potsdam, Germany

**Keywords:** Exercise intensity, Intensity distribution, Vagal threshold, Endurance training, Performance testing

## Abstract

**Background:**

Exercise intensities are prescribed using specific intensity zones (moderate, heavy, and severe) determined by a ‘lower’ and a ‘higher’ threshold. Typically, ventilatory (VT) or blood lactate thresholds (LT), and critical power/speed concepts (CP/CS) are used. Various heart rate variability-derived thresholds (HRVTs) using different HRV indices may constitute applicable alternatives, but a systematic review of the proximity of HRVTs to established threshold concepts is lacking.

**Objective:**

This systematic review aims to provide an overview of studies that determined HRVTs during endurance exercise in healthy adults in comparison with a reference VT and/or LT concept.

**Methods:**

A systematic literature search for studies determining HRVTs in healthy individuals during endurance exercise and comparing them with VTs or LTs was conducted in Scopus, PubMed and Web of Science (until January 2022). Studies claiming to describe similar physiological boundaries to delineate moderate from heavy (HRVTlow vs. VTlow and/or LTlow), and heavy from severe intensity zone (HRVThigh vs. VThigh and/or LThigh) were grouped and their results synthesized.

**Results:**

Twenty-seven included studies (461 participants) showed a mean difference in relative HR between HRVTlow and VTlow of − 0.6%bpm in weighted means and 0.02%bpm between HRVTlow and LTlow. Bias between HR at HRVTlow and VTlow was 1 bpm (limits of agreement (LoA): − 10.9 to 12.8 bpm) and 2.7 bpm (LoA: − 20.4 to 25.8 bpm) between HRVTlow and LTlow. Mean difference in HR between HRVThigh and VThigh was 0.3%bpm in weighted means and 2.9%bpm between HRVThigh and LThigh while bias between HR at HRVThigh and VThigh was − 4 bpm (LoA: − 17.9 to 9.9 bpm) and 2.5 bpm (LoA: − 12.1 to 17.1 bpm) between HRVThigh and LThigh.

**Conclusion:**

HRVTlow seems to be a promising approach for the determination of a ‘lower’ threshold comparable to VTlow and potentially for HRVThigh compared to VThigh, although the latter needs further empirical evaluation. LoA for both intensity zone boundaries indicates bias of HRVTs on an individual level. Taken together, HRVTs can be a promising alternative for prescribing exercise intensity in healthy, male athletes undertaking endurance activities but due to the heterogeneity of study design, threshold concepts, standardization, and lack of female participants, further research is necessary to draw more robust and nuanced conclusions.

**Supplementary Information:**

The online version contains supplementary material available at 10.1186/s40798-023-00607-2.

## Key points

- The present systematic review is the first one that systematically compares methods of intensity zone determination derived from heart rate variability (HRVTlow/HRVThigh) with more traditional concepts derived from ventilatory measurements (VTlow/VThigh) and blood lactate concentration (LTlow/LThigh).
- The findings of our data synthesis reveal that HRVTs for healthy, male adults participating in endurance-type activities show in general a high overall correlation but a very heterogeneous level of agreement with established VTs and/or LTs as the agreement strongly varies with the chosen reference method and/or HRVT approach.
- HRVTlow seems a promising approach for the determination of a ‘lower’ threshold comparable to VTlow that could be used to demarcate the boundary between moderate and heavy exercise intensity. This may also be the case for HRVThigh compared to VThigh to denote the boundary between the heavy and severe exercise domain but needs further evaluation. Limits of agreement denote potential bias on an individual level.
- HRVT validation studies used very heterogeneous methodologies, which limit the comparability of results. Evidence for female athletes is scarce, and for some HRVT-approaches not available at all. Thus, more high-quality research in this direction is urgently needed.

## Background

The training intensity distribution framework divides exercise intensity into predetermined zones and plays a crucial role in training monitoring for both performance enhancement and health preservation of individuals performing endurance exercise [1, 2]. In general, these exercise intensity zones are discrete domains defined by internal (e.g., ventilatory/metabolic/cardiorespiratory/perceptive) and/or external (e.g., power/speed) load indices that aim to demarcate exercise-induced homeostatic perturbations and delineate a gradual transition from steady-state to non-steady-state organismic functioning [3, 4]. Within the exercise intensity distribution model of endurance sports, at least three zones are typically used [1, 5, 6]. These zones are traditionally inferred from “threshold-like” approaches in accordance with physiologic boundaries of internal load measures such as blood lactate concentration and/or ventilatory/gas exchange values [5, 7–9].

### Exercise Intensity Zones: ‘Traditional’ Concepts in Science and Practice

Zone 1 typically represents a moderate exercise intensity which induces a plateau in VO_2_ with blood lactate concentrations close to baseline that are indicative of predominantly oxidative re-phosphorylation of ATP. Zone 2 characterizes the heavy intensity zone in which a ‘slow component’ and a delayed steady state of VO_2_, as well as a rise in blood lactate above baseline that stabilizes over time are present, while zone 3 corresponds to a severe exercise intensity with the ‘VO_2_-slow component’ that drives towards VO_2max_ in accordance with the continuous and substantial increase in blood lactate that exceeds lactate clearance capabilities [1, 5, 8, 9]. Traditional approaches to the determination of exercise intensity zones use physiological thresholds that are based on blood lactate [4, 9, 10] and ventilatory measurements [7]. Within this context, a large body of threshold concepts evolved and has been the subject of a vibrant scientific debate for more than 50 years [4, 8]. From a practical point of view, a ‘first (lower)’ and a ‘second (higher) threshold’ are typically used to differentiate between the three exercise intensity domains [4, 11] and provide the opportunity to determine the amount of time spent within a specific zone (i.e., training intensity distribution) [6, 12]. In this context, the most frequently applied threshold concepts in training practice demarcate zones 1 and 2 based on the first lactate threshold (LT1), the gas exchange threshold (GET) and/or a (first) ventilatory threshold (VT1/VT) [1, 5, 6, 9]. In addition, zones 2 and 3 are commonly determined by the respiratory compensation point (RCP), the maximal lactate steady state (MLSS) or a second lactate threshold (LT2) as a proxy for MLSS [1, 5, 9]. However, given the fact that there is a large heterogeneity of (i) testing protocols (e.g., slope, stage duration and increment of graded exercise or ramp tests) and (ii) determination methods (e.g., linear vs. nonlinear regression models, amount of data points used, fixed or variable threshold concepts), as well as (iii) a plethora of definitions and names of thresholds (e.g., blood lactate: LT/LT1, LT2, (individual) anaerobic threshold, MLSS; ventilatory: VT/VT1/GET, VT2/RCP), a general confusion within the scientific and practical debate on threshold concepts in exercise science is still present [8]. Most recent views on exercise intensity prescription point towards the primary use of LT/GET (and VT) for the demarcation of the boundary of moderate to heavy exercise intensity domain, while the separation of heavy and severe exercise intensity might be best determined using the critical power/speed (CP/CS) concept [4, 8, 10, 13, 14]. The latter concept of CP/CS uses the hyperbolic power/speed–duration relationship to define exercise tolerance for endurance exercise and likely represents the threshold above which there is a continuous obligatory glycolytic contribution with substantial net lactate accumulation making it well-situated to differentiate between heavy and severe exercise intensity [8, 14–16]. However, besides the ongoing debate on ‘traditional’ threshold concepts, the ‘results-proven training practice’ still uses a large variety of VT and/or LT concepts to yield training intensity distribution boundaries [6, 17]. Additionally, threshold concepts based on, e.g., concentration changes of deoxygenated hemoglobin and myoglobin determined by muscle oximetry [18], as well as on heart rate (HR) [19, 20] and HR variability (HRV) [21–23], have been developed and evaluated in numerous method comparison studies.

### Heart Rate Variability Thresholds (HRVT): Perspectives for Exercise Intensity Prescription

In this context, especially HRV thresholds (HRVT) have shown promise as an applicable and economical way to determine exercise intensity zones [23, 24], because HRV can be tracked noninvasively and continuously in real time by relatively inexpensive and miniaturized wearable devices (e.g., chest belt connected with a smartwatch or smartphone application) [25, 26]. Generally, changes in HRV with increasing exercise intensity mirror the complex interplay between parasympathetic withdrawal, concomitant rise in sympathetic activity, and other related (non-neural) factors [21, 27–29]. Therefore, comparable to traditional threshold concepts, HRVTs aim to capture fundamental ‘tipping points’ of complex neuro-autonomic regulation processes by using specific time, frequency/time–frequency and/or nonlinear HRV-metrics to determine a first, rather lower (HRVTlow) and a second, higher (HRVThigh) threshold that, in turn, allow differentiation of moderate from heavy and heavy from severe exercise intensity, respectively [30]. Specifically, HRVTlow methods are mainly characterized by the rapid reduction of HRV indices from rest to moderate exercise intensity with a subsequent minimum or plateau around 50–60% of maximal oxygen uptake [23]. This transitional process can be captured by several linear HRV parameters in time and frequency domain. In particular, indices reflecting parasympathetic activity such as the root mean square of successive differences (RMSSD), standard deviation one of Poincaré Plot analysis (SD1) or high-frequency power (HF, typically 0.15–0.4 Hz) of spectral analysis may be suitable indices [23, 31–33]. Hence, different methodological approaches including several time and frequency-based analysis methods were developed to identify HRVTlow that corresponds to traditional approaches of blood lactate (LTlow) and/or ventilatory/gas exchange thresholds (VTlow) [23, 34, 35]. From a physiological perspective, the strong reduction of time and frequency indices of HRV during moderate exercise with a minimum or plateau around 50–60% of maximal oxygen uptake [23] seems to appear just before the onset of blood lactate accumulation and an increase in minute ventilation due to a corresponding excess of CO_2_ [23, 35, 36], which suggests a common involvement of higher cardiovascular and metabolic control systems [37]. Moreover, these findings imply that direct physiological links between feed-forward mechanisms from higher brain centers and feedback mechanisms from muscle mechanoreceptors exist that drive, on the one hand, initial vagal withdrawal as well as an initial reduction in cardiac sympathetic neural activity due to loading of the cardiopulmonary baroreceptors [38] and, on the other hand, metabolic/ventilatory changes defining the transition from moderate to heavy exercise intensity.

With a further increase in exercise intensity, a second threshold-like behavior of HRV (HRVThigh) has been observed by time–frequency analysis and displays an abrupt re-increase in high-frequency power (HF), peak frequency of HF (HFpeak) or a product of both when plotted against work rate [21, 22, 36]. This physiological observation seems to be directly related to the disproportional rise in breathing frequency once the exercise intensity corresponds to a second ventilatory ‘tipping point’ (VThigh)—in particular, when the boundary of heavy-to-severe exercise is exceeded [36]. Theoretically, the observed re-rise in indices of (time-) frequency analysis is probably driven by a complex physiological interplay of mechanisms of vagal withdrawal and sympathetic activation that are related to (i) muscle mechano- and/or metaboreceptors and an increased central command, (ii) a concomitant increase of the mechanical influence of venous return on the stretch of the sinus node caused by elevated breathing frequency and/or volume and muscle pump as well as a cardio-respiratory-locomotor coupling, and (iii) the increase of circulating catecholamines [36, 38, 39]. Regarding the frequency content of HRV during non-stationary exercise conditions, it is noteworthy that traditional spectral methods like the fast Fourier transform (FFT) or autoregressive modeling (AR) show conflicting results and thus may not be reliable approaches to determine a HRVThigh [28]. In this context, time–frequency methods such as short-time Fourier transformation [39] and smoothed pseudo-Wigner–Ville distribution [40] seem to be more promising approaches as these methods may properly track instantaneous changes in the HRV frequency content. In addition, an extension of the HF-band up to maximum breathing frequency or 1–2 Hz seems necessary when heavy to severe exercise intensities are involved because in this particular case the breathing frequency typically exceeds the upper HF-boundary (0.4 Hz) of resting conditions to a considerable extent [28, 30]. In addition to spectral approaches to HRVThigh, the aforementioned re-increase of HRV fluctuations has also been reported for Poincaré Plot analysis using the standard deviation two (SD2) [41, 42], which displays the dispersion of points along the line-of-identity of the plot and indicates the level of long-term variability in the HRV signal. However, regarding all HRVT approaches that are based on time and (time-)frequency domain it has to be considered that the magnitudes of all linear HRV markers above intensities of 50–60% of maximal oxygen uptake are low to very low and therefore strongly reduce signal-to-noise ratio, which may influence both the validity and reliability of HRVT assessment [30].

In addition to linear HRV time- and frequency-domain analysis, recent exercise studies have used methods of nonlinear dynamics to further elucidate complex cardiovascular regulation and to overcome some of the drawbacks of linear HRV analysis [43–45]. Among these methods, detrended fluctuation analysis (DFA) [46], recurrence quantification analysis (RQA) [47], sample entropy (SampEn), and compression entropy (CEn) [45, 48, 49] have been used for HRVT detection. These nonlinear methods have strong origins in signal theory and evaluate complex dynamics, regularity, and self-similarity of the HRV signal and display corresponding interrelations of underlying physiological regulation processes, rather than quantify HRV signal amplitude and frequency content [43, 49, 50]. In this regard, recent studies demonstrated that these methods are promising to demarcate exercise intensity transitions by identifying breakpoints and/or saturation behavior of HR dynamics [24, 51–54]. In particular, the short-term scaling exponent alpha1 of DFA (DFAa1) exhibits a broad dynamic range from moderate to severe exercise intensities [43, 55] and has a great potential for HRVT detection using fixed DFAa1 values of 0.75 and 0.5 that show moderate-to-high correlations with VTlow/LTlow and VThigh/LThigh [24, 51, 56], respectively. From a signal theory perspective, DFAa1 approaches to HRVT track the intensity-dependent loss of HRV correlation properties from the trade-off point between fractional Brownian motion and fractional Gaussian noise (1.0) occurring at moderate intensities over a half-way loss of correlation properties (0.75) that coincides with the transition to heavy exercise, towards uncorrelated/stochastic (0.5) or anti-correlated (< 0.5) HR dynamics at severe intensities [49, 55, 57–60]. From a physiological point of view, these changes in correlation properties are possibly caused by changes in the coupling strength and interaction of higher-order regulatory processes in the central autonomic network [37] that integrate the antagonistic interaction of vagal withdrawal and sympathetic activation [30, 43, 49], intracardiac biochemical changes and/or coupling mechanisms of different other cardiorespiratory and metabolic pathways [57], and feedback from muscle mechano-/-metaboreceptors [38]. Thus, changes in correlation properties of HR dynamics could be a promising tool for displaying the regulation quality of common cardiovascular and metabolic control systems [43].

Taken together, several linear and nonlinear HRVT methods using time, (time)–frequency and nonlinear domain measures have been utilized to demarcate exercise intensity zones based on the complex interplay of vagal withdrawal, increased sympathetic activity and other non-neural factors. In this regard, numerous studies investigated whether HRVTs are comparable to traditional threshold concepts based on VT and LT measurements that denote the transition from moderate to heavy (VTlow/LTlow) and heavy to severe (LThigh/VThigh) exercise, respectively. However, the current state of the literature in this research field has not been systematically evaluated. A lack of a systematic analysis of the available evidence on the capability of HRVT concepts to reflect commonly used threshold approaches can impede progress in both research and the practical application of HRVT concepts. Therefore, the aim of this review is to provide a systematic overview of all studies that determined HRVTs in healthy adults during endurance exercise and compared HRVTs to most common concepts using blood lactate- and/or ventilatory-derived thresholds. As far as possible, recommendations for specific settings will be given, which may help assist sports practitioners and scientists when using HRVT approaches to performance testing or prescription of exercise intensity zones.

## Methods

### Search Strategy

The systematic review was conceptualized and carried out in July–September 2021 (with the last update conducted in January 2022) according to the guidelines for the Preferred Reporting Items for systematic Reviews and Meta-Analyses (PRISMA) [61] and registered at Open Science Framework (OSF) (https://osf.io/z63wv/). The electronic databases searched included Scopus, PubMed and Web of Science (with no restriction concerning publication date), and the following search string was used: (HRV OR heart rate variability OR autonomic nervous system) AND (threshold OR zone) AND (endurance OR exercise OR running OR cycling). This search string allows to find studies involving various endurance-type exercise modalities.

### Inclusion and Exclusion Criteria

**Fig. 1:**
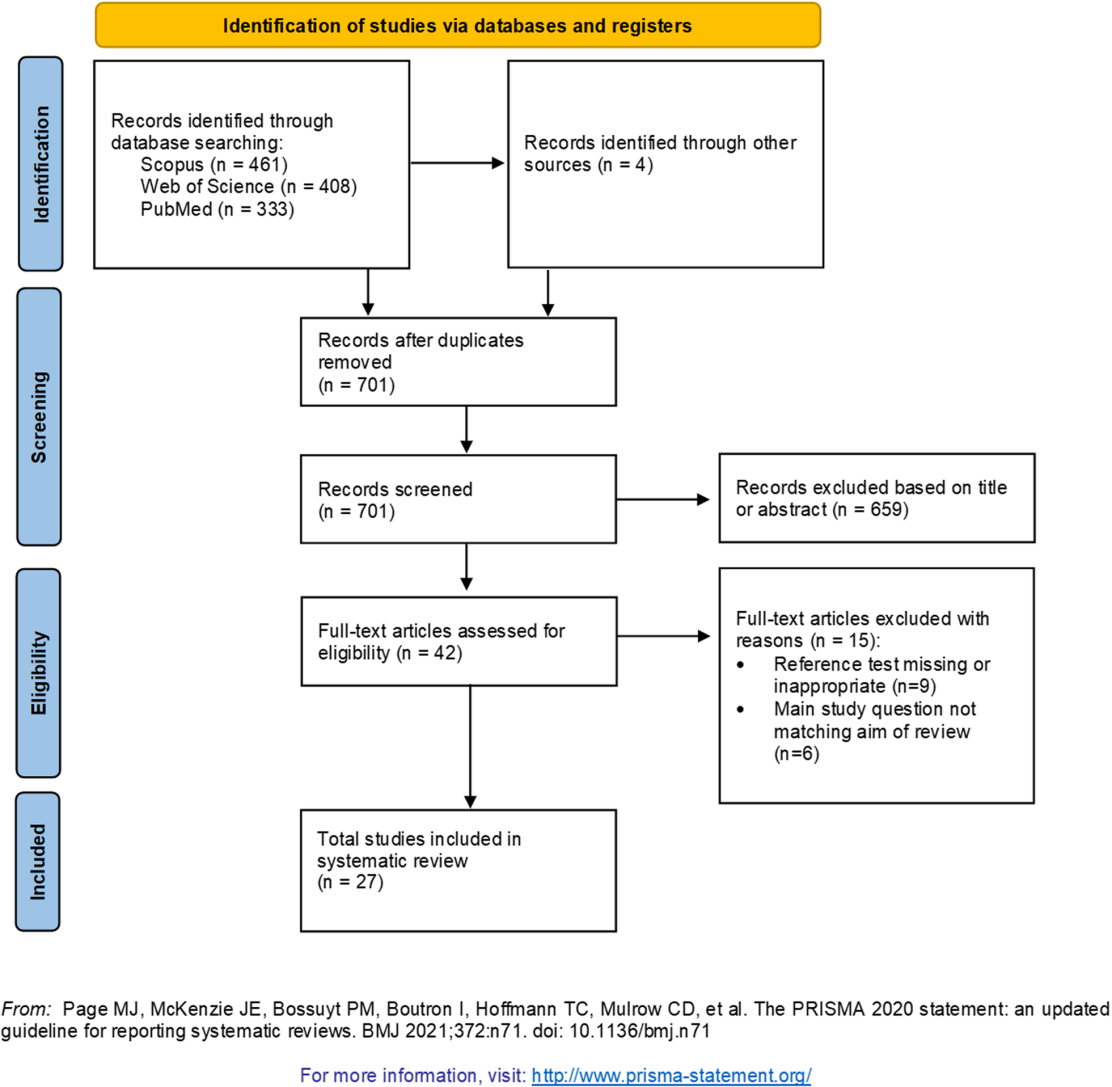
Flow chart of article selection process from article identification using a search string, over screening and eligibility testing to inclusion in the review. Number of studies is displayed as *n*

This systematic review aims to identify and group all scientific studies that assessed HRVT exercise zone boundaries and compared HRVTs with threshold concepts derived from ventilatory (VT) and/or blood lactate (LT) measurements. To identify eligible studies, we followed the PICOS-principle (“PICOS” stands for participants (P), intervention (I), comparisons (C), outcomes (O), and study design (S)) [62, 63]. Accordingly, we included all studies that met the following criteria: (P) only studies dealing with healthy adults (> 18 years) regardless of age or performance level are considered as relevant, (I) we applied all endurance-type cyclic movements (e.g., running, cycling, cross-country skiing, swimming) during an incremental or graded exercise test concerning the intervention, (C) all considered studies needed to compare HRV-derived thresholds (HRVTlow and/or HRVThigh) with commonly used threshold approaches using ventilatory (VTlow and/or VThigh) or/and blood lactate (LTlow and/or LThigh) parameters, (O) we considered all studies as relevant that assessed time domain, (time-)frequency domain and/or nonlinear domain HRV metrics, and (S) no specific restrictions in study design were applied. In addition to the PICOS principle, our search was limited to original articles published in peer-reviewed journals and written in English. References being cited by the retrieved articles were also examined for potential relevance. Conference abstracts, dissertations, theses, and other non-peer-reviewed articles were excluded. Figure 1 illustrates the screening and selection process employed.

### Study Selection

In the first step, all duplicates were removed by the first author (SK). In the second step, a title—and abstract—screening was performed by two independent reviewers (SK and TG) to identify eligible studies. Afterward, the full texts of remaining studies were screened by the same reviewers and 15 studies were excluded with reason (see Fig. 1 for a detailed overview). In a subsequent step, the reference lists of the relevant studies were searched for additional publications not having been identified during the electronic database search but meeting our inclusion criteria. In case of a disagreement between the two reviewers, a consensus was achieved by a discussion or input from a third author (OH). A flow chart illustrating the selection procedure is provided in Fig. 1.

### Data Extraction and Synthesis

From the relevant articles, the following information was extracted and entered in an Excel sheet (Microsoft Corporation. (2018). Redmont, WA, Microsoft Excel. Retrieved from https://office.microsoft.com/excel) by the first author (SK) and confirmed by another author (FH): (i) details of publication (i.e., authors, year, journal, publication date), (ii) characteristics of the study population (i.e., age, sample size, sex), (iii) study design (i.e., design of the incremental/graded exercise test, type of exercise, description of HRV measurements (incl. preprocessing) and threshold determination, information about validation with other threshold concepts), and (iv) study results.

As a major outcome of the included studies the mean ± standard deviation of the HR at the respective threshold are reported. Based on these values, Cohen’s *d* was calculated to display an effect size as additional indicator of difference between means. Cohen’s *d* was calculated based on [64, 65]. In addition, bias as mean difference between HRVT and reference threshold (VT or LT) was extracted from Bland and Altman analyses [66], as well as lower and upper limits of agreement. If HR data are not provided in the main manuscript or in Additional file 1, running speed, power and/or oxygen uptake at the respective threshold are reported instead (reported parameters depend on the availability/reporting of data in the original study). Additionally, if available, correlation coefficients between performance indicators at each threshold were extracted. If a study experimented with different HRVT methods, the one with the best agreement was included in the synthesis.

Further, a synthesis was generated from the extracted data from the included studies. Firstly, weighted pooling of systematic error (bias) and standard deviation was conducted for HR and/or power/running speed at the specific thresholds, respectively. Since not all studies provided data on absolute reliability (i.e., Bland and Altman analyses), additionally weighted mean and standard deviations for HR, power and running speed at HRVTs and/or VTs, LTs were determined. This weighted mean is reported as difference in percent of the mean result of the reference test. Finally, a weighted Pearson correlation coefficient was calculated in the case that the original study reported correlation coefficients. Due to the large heterogeneity concerning data presentation in the included studies, we had to refrain from conducting further meta-analytical calculations. All statistical tests were deemed to be statistically significant at *p* ≤ 0.05 and are presented as mean ± standard deviation. Effect sizes were denoted by Cohen’s *d* (low: 0.2 < *d* < 0.5, moderate: 0.5 < *d* < 0.8, high: 0.8 < *d* [65]. The weighted Pearson correlation coefficients (*r*) were classified according to recommendations for reliability measures [67].

### Assessment of Methodological Quality of Included Studies

The methodological quality of the included studies was assessed using the revised tool Quality Assessment of Diagnostic Accuracy Studies (QUADAS2) [68]. The risk of bias and applicability concerns for the review question were independently assessed by two authors (SK and OH). Due to the complexity of HRV methodology, we also rated the methodological quality by using a HRV-specific tool (Standard for Reporting Diagnostic Accuracy Studies (STARD_HRV_, Table 1 [69]), which is a modified version of the original STARD [70]. The methodological quality assessment by the means of STARD_HRV_ was performed by two authors (SK and OH), and STARD_HRV_ was slightly adapted in items 1, 9, 19 and 21, see Additional file 1: Table S1, to best fit the purpose of this systematic review. Any disagreement concerning the ratings of methodological quality was resolved by consensus or a discussion with a third author (TG).

**Table 1:** Overview of study results using the domains HRV-index test, LT and/or VT reference test, graded exercise test protocol, participants and results. Note that only values and methods included in the synthesis are reported, as some studies evaluated more than one HRV threshold concept of which the one with the best agreement was then chosen for the synthesis. In the results section the available information on test results, Bland and Altman indices and correlation analysis are presented, respectively. Additionally, effects sizes are denoted as Cohen's *d*, further confidence intervals (CI), means and standard deviations of values at ventilatory thresholds (VTlow/VThigh), and/or lactate thresholds (LTlow/LThigh) and heart rate variability thresholds (HRVTlow/HRVThigh) are reported

Study	Index test	Reference test	GXT protocol	Participants	Results
Anosov et al. 2000 [34]	HRVTlow: Hilbert-transform (time–frequency method) using first HF rise after low plateau	VTlow: GET using VCO2/VO2 (V-slope) method	Cycling ergometer, increase 20 W every min	22 (13f, 9m) untrained participants	2–14% difference at VTlow and HRVTlow
Blain et al. 2005 [39]	HRVTlow: STFT (time–frequency method) using first fRSA increase HRVThigh: STFT (time–frequency domain) using second fRSA increase	VTlow: VT1 using VE and VE/VO2 method; VThigh: VT2 using VE and VE//VCO2 method	Cycling ergometer, increase 37.5 W every 2 min	14 sedentary men, 12 male endurance athletes	Sedentary subgroup, HF—151 ± 19.5 W at VTlow and 150.3 ± 18.7 W at HRVTlow (*d* = − 0.037; CI − 0.916 to 0.843), 200.3 ± 29.4 W at VThigh and 198.3 ± 28.8 W at HRVThigh (*d* = − 0.069; CI − 0.948 to 0.811) Endurance athletes: 247 ± 33.6 at VTlow and 247.3 ± 32.8 W at HRVTlow (*d* = 0.009; CI − 0.941 to 0.959), 310.9 ± 26.7 W at VThigh and 316 ± 28.8 W at HRVThigh (*d* = 0.184; CI − 0.768 to 1.135)
Cassirame et al. 2015 [96]	HRVTlow: STFT (time–frequency method) using first HF*pHF increase HRVThigh: STFT (time–frequency domain) using second HF*pHF increase	VTlow: VT1 using VE and VE/VO2 method; VThigh: VT2 using VE and VE/VCO2 method	ski-mountaineering on an alpine slope, increase 0.5 km/h every min	9 (4f, 5m) competitive ski-mountaineers	HRVTlow could only be determined for one participant, bias and standard deviation of VThigh versus HRVThigh − 0.02 bpm and 1.87 bpm, *r*^2^ from linear regression between HR at VThigh and HRVThigh was 0.91
Cottin et al. 2007 [40]	HRVTlow: SPWVD (time–frequency method) using first HF*pHF increase HRVThigh: SPWVD (time–frequency domain) using second HF*pHF increase	VTlow: VT1 using first nonlinear increase VE/VO2 while VE/VCO2 remains constant VThigh Using nonlinear increase in VE/VCO2 and second increase in VE/VO2	Track running, increase 0.5 km/ every min	12 professional male soccer players	Speed at VTlow was 9.83 ± 1.12 km/h and 10.08 ± 1.29 km/h (*d* = 0.207; CI − 0.745 to 1.159) while speed at VThigh was 12.55 ± 1.31 km/h and 12.58 ± 1.33 km/h (*d* = 0.023; CI − 0.927 to 0.972), bias between VTlow and HRVTlow was − 0.25 km/h and − 0.05 km/h between VThigh and HRVThigh, correlation coefficients for HR at VTlow and HRVTlow and VThigh and HRVThigh were *r* = 0.97 and *r* = 0.98
Cottin et al. 2006 [36]	HRVTlow: STFT (time–frequency method) using first HF and HF*pHF increase HRVThigh: STFT (time–frequency domain) using second HF*pHF increase	VTlow: VT1 using first nonlinear increase VE/VO2 while VE/VCO2 remains constant VThigh: Using nonlinear increase in VE/VCO2 and second increase in VE/VO2	Cycling ergometer, 15–20 W every min	11 competitive male cyclists and triathletes	Power at VTlow was 219 ± 45 W and 220 ± 48 W at HRVTlow (*d* = 0.021; CI − 0.971 to 1.013) and 293 ± 45 W at VThigh and 294 ± 48 W at HRVThigh (*d* = 0.021; CI − 0.971 to 1.013), bias between VTlow and HRVTlow was 0.45 W and 0.91 W between VThigh and HRVThigh, correlation coefficients for HR at VTlow and HRVTlow and VThigh and HRVThigh were *r* = 0.97 and *r* = 0.98
Cunha et al. 2014 [87]	HRVTlow: HRVT using SD1 less than 3 ms (time domain)	VTlow: GET using modified V-slope first increase in VO2/VCO2	Cycling ergometer, running and walking tests on a treadmill	16 regularly active male students	HR in cycling, walking and running was 133 ± 7 bpm, 141 ± 11 bpm and148 ± 14 bpm at VTlow and 135 ± 9 bpm, 144 ± 16 bpm and 152 ± 15 bpm at HRVTlow (*d* = 0.248; CI − 0.578 to 1.074; *d* = 0.219; CI − 0.606 to 1.043; *d* = 0.276; CI − 0.551 to 1.102), bias between HR at VTlow and HRVTlow ranged from 2 to 4 bpm with standard deviation ranging from 3 to 8 for cycling, walking and running, correlation coefficients for HR at VTlow and HRVTlow for cycling walking and running were *r* = 0.93, *r* = 0.92 and *r* = 0.93
Di Michele et al. 2012 [90]	HRVThigh: AT-HRV using discrete step before first increase in HF power / HF power-RSA or increase associated with highest speed if 2 increses occurred (time–frequency domain)	LThigh: AT using lactate turnpoint determined as discrete step immediately before the observation of a sudden and sustained increase	7 × 200 m swimming test with individualized step protocol	14 (8f, 6m) high-level swimmers	HR at LThigh was 182 ± 8.1 bpm and 181.1 ± 8.2 bpm at HRVThigh (*d* = − 0.11; CI − 0.99 to 0.77), bias between HR at VThigh and HRVThigh was − 0.9 bpm with a standard deviation of 2.95 bpm
Dourado et al. 2010 [110]	HRVTlow: HRVT using the first point in which the difference in SD1 is less than 1 ms in 2 consecutive stages (time domain)	VTlow: VT (Wassermann) using VE/VO2 and VE/VCO2	Shuttle walk test, increase 0.6 km/h every minute	10 (3f, 7m) sedentary participants	Speed at VTlow was 5.04 ± 1 km/h and 5.1 ± 1.04 km/h at HRVTlow (*d* = 0.059; CI − 0.821 to 0.938), bias in oxygen uptake between VTlow and HRVTlow was − 0.05 l/min with a standard deviation of 0.13 l/min
Dourado & Guerra 2013 [111]	HRVTlow: HRVT using the first point in which the difference in SD1 is less than 1 ms in 2 consecutive stages (time domain)	VTlow: VT using first nonlinear increase in VE/VO2 while VE/VCO2 remains constant	Shuttle walk test, increase 0.6 km/h every minute	31 (17f, 14m) healthy participants	Bias between VTlow and HRVTlow was 0.04 km/h with a standard deviation of 0.67 km/h, ICC for speed at VTlow and HRVTlow was *r* = 0.92
Garcia-Manso et al. 2013 [97]	HRVTlow: Aerobic (HRV) threshold using the first increase of frequency peaks of the high frequency-very high frequency band HRVThigh: Anaerobic (HRV) threshold using the second increase of the frequency peaks curve (time–frequency domain)	VTlow: VT1 by visual detection using VE/VCO2 (ventilatory equivalent CO2), VE/VO2 (ventilatory equivalent O2), PETCO2 (end-tidal partial pressure CO2), and PETO2 (end-tidal partial pressure O2) VThigh: VT2 based on Gaskill, Ruby [73]	Cycling ergometry, increase 25 W every minute	8 high-performance male cyclists	Bias between VTlow and HRVTlow was 1.88 W with a standard deviation of 5.26 W, bias between VThigh and HRVThigh was − 4.38 W with a standard deviation of 12.04 W
Garcia-Tabar et al. 2013 [91]	HRVTlow: SD1Tlow using SD1 value 1 ms above minimal SD1 (time domain)	LTlow: AeT using basic lactate + 0.2 mmol/l	Cycling ergometry, increase 58 W every 3 min	12 world-class male cyclists	HR at LTlow was 132.7 ± 9.6 bpm and 129.6 ± 10.4 bpm at HRVTlow (*d* = − 0.31; CI − 1.265 to 0.646), bias expressed as relative power between LTlow and HRVTlow was 0.15 W/kg with a standard deviation of 0.29 W/kg, correlation coefficient for relative power at LTlow and HRVTlow was *r* = 0.88
Granell & De Vito 2018 [112]	HRVTlow: THFp using the stabilization point of frequency peak of high frequency defined as first point where no significant further change appears (time–frequency domain)	VTlow: VT using V-slope	Cycling ergometry, increase 20 W every min	10 regularly active men	Bias between VTlow and HRVTlow was 5 bpm with a standard deviation of 7.65 bpm
Hamdan et al. 2016 [54]	HRVThigh: Cardiac vagal threshold using minimum of polynomial third order fit of H_C_ time course (nonlinear domain)	LThigh: Aerobic threshold according to Dickhuth, Röcker [94]	Cycling ergometry, increase 50 W every 3 min	19 healthy male students	Power at LThigh was 184 ± 28 W and 192 ± 34 W at HRVThigh (*d* = 0.257; CI − 0.501 to 1.015), bias between LThigh and HRVThigh was 7.95 W with a standard deviation of 18.08 W, correlation coefficient for power at LThigh and HRVThigh was *r* = 0.86
Karapetian et al. 2008 [35]	HRVTlow: HRVT (SD) using SD1 and point of no further decline (time domain)	LTlow: LT using visually determination at first increase from low-intensity exercise VTlow: VT using modified V-slope [73]	Cycling ergometry, increase 25 W every 3 min	24 (15f, 9m) regularly active participants	Bias between VTlow and HRVTlow expressed in oxygen consumption was 0.065 l/min and 0.05 l/min between LTlow and HRVTlow with standard deviations of 0.47 l/min and 0.46 l/min, respectively, correlation coefficient for VO2 at HRVTlow and LTlow was *r* = 0.82 and for VO2 at HRVTlow and VTlow *r* = 0.89
Mankowski et al. 2016 [42]	HRVThigh: HRVT2 using visual inspection of nRMSSD minimum (time domain)	VThigh: VT2 using the second nonlinear increase in VE/VCO2	Cycling ergometry, increase 12–18 W every min	11 (3f, 8m) regularly active participants	Power at VThigh was 251.3 ± 44.7 W and 243.6 ± 44.2 W at HRVThigh (*d* = − 0.173; CI − 1.167 to 0.821), bias between VThigh and HRVThigh was 4.9 W with a standard deviation of 20.35 W
Mateo-March et al. 2022 [56]	HRVTlow: HRVT1 using DFAa1 = 0.75 HRVThigh: HRVT2 using DFAa1 = 0.5 (nonlinear domain)	LTlow: LT1 using blood lactate increase above baseline LThigh: LT2 using increase of over 2 mmol/l above baseline	Cycling ergometry, increase 25 W every min	38 male elite cyclists	HR at LTlow 153 ± 14 bpm and 150 ± 17 bpm at HRVTlow (*d* = 0.02; CI − 0.30 to 0.34), CI for bias between LTlow and HRVTlow were − 1.62 bpm to 6.99 bpm with CI for limits of agreement ranging from 20.94 to 35.80 bpm and − 30.43 to − 15.58 bpm for upper and lower limits of agreement, respectively, HR at LThigh was 176.84 ± 11.35 bpm and 173.18 ± 12.32 bpm at HRVThigh (*d* = 0.41, CI 0.07–0.74), CI for bias between LThigh and HRVThigh were 0.69 bpm to 6.77 bpm with CI for limits of agreement ranging from 16.34 to 26.81 bpm and − 19.35 to − 8.88 bpm for upper and lower limits of agreement, respectively
Mendia-Iztueta et al. 2016 [86]	HRVTlow: HRVT1 visual determination using point of no further decrease of SD1 (time domain), HRVThigh: HRVT2 visual determination using high frequency power and high frequency power considering RSA point of last increase (time–frequency domain)	VTlow: VT1 from VE/VO2 and VCO2/VO2 VThigh: VT2 using VE/VO2 and VE/VCO2	Five different roller-skiing techniques on a treadmill, increase every 3 min (mix of velocity and incline depending on technique)	10 (5f, 5m) national level cross-country skiers	In five different activities (diagonal striding, two skating rhythms, double poling and Nordic walking) bias in HR between VTlow and HRVTlow ranged from − 9 to 9 bpm with standard deviations of 6.63 to 12.75 bpm and bias between VThigh and HRVThigh ranged from − 18 to − 1 bpm with standard deviations of 3.06 to 18.62 bpm
Mourot et al. 2014 [89]	HRVThigh: VT2 from HRV method 4 using point before increase in high frequency, high frequency considering RSA and considering locomotion (time–frequency domain)	VThigh: VT2 using second increase in VE with concomitant increase in VE/VO2 and VE/VCO2	Roller-skiing on a treadmill, increase 0.3 km/h and 1% gradient every min	16 trained male ski-mountaineers	Power at VThigh was 186.4 ± 6.7 bpm and 185.1 ± 6.8 bpm at HRVThigh (*d* = − 0.193; CI − 1.017 to 0.632), bias between VThigh and HRVThigh was 0.1 bpm with a standard deviation of 3.06 bpm, correlation coefficient for HR at VThigh and HRVThigh was *r* = 0.825
Nascimento et al. 2017 [41]	HRVTlow: HRVT1 using Dmax based on SD1 HRVThigh: HRVT2 using Dmax based on SD2 (time domain)	LTlow: LT1 using running speed at 2 mmol/l blood lactate value LThigh: LT2 using running speed at 3.5 mmol/l blood lactate value	Treadmill running, increase 1 km/h every 3 min	19 trained male long-distance runners	HR at LTlow was 155 ± 17 bpm and 155 ± 15 bpm at HRVTlow (*d* = 0.00; CI − 0.755 to 0.755) and 173 ± 12 bpm at LThigh and 173 ± 9 at HRVThigh (*d* = 0.00; CI − 0.755 to 0.755), bias between LTlow and HRVTlow expressed as running speed was − 0.26 km/h with a standard deviation of 2.07 km/h and bias between LThigh and HRVThigh was 0 km/h with a standard deviation of 1.29 km/h
Nascimento et al. 2019 [92]	HRVTlow: HRVT1 using Dmax based on SD1 HRVThigh: HRVT2 using Dmax based on SD2 (time domain)	LTLOW: LT1 using lowest value of lactate to speed ratio LTHIGH: LT2 using LT1 plus 1.5 mmol/l blood lactate	Treadmill running, increase 1 km/h every 3 min	19 trained male long-distance runners	HR at LTlow was 151 ± 14 bpm and 155 ± 15 bpm at HRVTlow (*d* = 0.276; CI − 0.483 to 1.034) and 173 ± 9 bpm at LThigh and 159 ± 15 at HRVThigh (*d* = − 1.132; CI − 1.945 to − 0.319), bias between LTlow and HRVTlow expressed as running speed was 0.84 km/h with a standard deviation of 1.45 km/h and bias between LThigh and HRVThigh was − 1.07 km/h with a standard deviation of 0.91 km/h
Park et al. 2014 [113]	HRVTlow: HF times fHFTW determination using intersection of trend lines based on data points below and above VT (time–frequency domain)	VTlow: VT using VE against power in W [99]	Cycling ergometry, increase 20 W every min	15 (8f, 7m) trained cyclists	Power at VTlow was 182.8 ± 38 W and 185.1 ± 50.3 W at HRVTlow (*d* = 0.052; CI − 0.798 to 0.901), bias between VTlow and HRVTlow expressed as power was − 2.4 W with a standard deviation of 23.1 W, correlation coefficient for power at VTlow and HRVTlow war *r* = 0.90
Queiroz et al. 2016 [114]	HRVTlow: HRVTSD1 using SD1 of less than 3 ms (time domain)	VTlow: VT using increase of VE/VO2 without increase of VE/VCO2	Cycling ergometry, increase 15 W every min	31 untrained healthy men	HR of the healthy sub-group at VTlow was 140.6 ± 14.53 bpm and 137.8 ± 6.32 bpm at HRVTlow (*d* = − 0.25; CI − 0.843 to 0.343), bias between oxygen consumption at VTlow and HRVTlow was 0.26 l/min with a standard deviation of 0.26 l/min
Ramos-Campo et al. 2018 [85]	HRVTlow: HRTV1 using point where the difference between SD1 values of 2 consecutive stages was less than 1 ms and no longer changed (time domain), HRVThigh: HRVT using final abrupt increase in peak high frequency (time–frequency domain)	VTlow: VT1 using first increase ov VE/VO2 over workload VThigh: VT2 using disproportionate increase in VCO2 over workload and respiratory exchange ratio greater 1	Treadmill running, increase 1 km/h every min	24 professional male basketball players	HR at VTlow was 142.5 ± 9.4 bpm and 140.1 ± 10.5 bpm at HRVTlow (*d* = − 0.241; CI − 0.915 to 0.433) and 173.5 ± 10.9 bpm at VThigh and 175.1 ± 11.5 bpm at HRVThigh (*d* = 0.143; CI − 0.53 to 0.815), bias between HR at VTlow and HRVTlow was 1.52 bpm with a standard deviation of 1.2 bpm and bias between HR at VThigh and HRVThigh was 1.16 bpm with a standard deviation of 0.87 bpm, correlation coefficients for HR at VTlow and HRVTlow and VThigh and HRVThigh were *r* = 0.57 and *r* = 0.90, respectively
Rogers et al. 2021 [24]	HRVTlow: HRVT using DFAa1 = 0.75 (nonlinear domain)	VTlow: VT1 using V-slope, VE/VO2 and excess CO2 [73] and PetO2	Treadmill running, increase 1.3 km/h and 2% gradient every 3 min	17 regularly active men	HR at VTlow was 152 ± 21 bpm and 154 ± 20 bpm at HRVTlow (*d* = 0.098; CI − 0.701 to 0.896), bias in HR between VTlow and HRVTlow was − 1.9 bpm with a standard deviation of 5.3 bpm, correlation coefficient for HR at VTlow and HRVTlow was *r* = 0.75
Rogers et al. 2021 [51]	HRVThigh: HRVT2 using DFAa1 = 0.5 (nonlinear domain)	VThigh: VT2 associated HR was determined using Oxynet http://oxynetresearch.promfacility.eu	Treadmill running, increase 1.3 km/h and 2% gradient every 3 min	17 regularly active men	HR at VThigh was 174 ± 12 bpm and 171 ± 16 bpm at HRVThigh (*d* = − 0.212; CI − 1.012 to 0.588), bias in HR between VThigh and HRVThigh was − 4 bpm with a standard deviation of 10.2 bpm, correlation coefficient for HR at VThigh and HRVThigh was *r* = 0.78
Shiriashi et al. 2018 [93]	HRVTlow: HRVT using coefficient of component variance (CCV) L/H elevated to a value of > 0.1, following a decrease to a value of < 5 ms2 in high frequency continuously for 60 s (frequency domain)	VTlow: VT using V-slope, VE/VO2 and excess CO2 [73] LTlow: LT using visual inspection based on first increase of blood lactate above baseline	Cycling ergometry, increase 20 W every min	30 (9f, 21m) healthy participants	Relative oxygen consumption at VTlowwas 23.3 ± 5.3 ml/kg/min and 23.7 ± 3.7 ml/kg/min at LTlow (*d* = 0.088; CI − 0.513 to 0.688) and 23.3 ± 5.7 ml/kg/min at HRVTlow (*d* = − 0.182; CI − 0.784 to 0.42) (*d* = 0.291; CI − 0.312 to 0.895), correlation coefficients between VTlow and HRVTlow and LTlow and HRVTlow were *r* = 0.921 and *r* = 0.853, respectively
Stergiopoulos et al. 2021 [98]	HRVThigh: HRVT2 determined as point of first abrupt increase of high frequency product after having reached the minimum (time–frequency domain)	VThigh: VT2 using the concomitant breakaway of VE/VO2 and VE/VCO2	Treadmill running, increase 0.5/h every min and 1 min protocol of Leger et al. 1988 [101]	15 male soccer players	Running speed at VThigh from the treadmill test was 12 ± 1 km/h and 12 ± 0.9 km/h at HRVThigh (*d* = 0.0; CI − 0.849 to 0.849), speed at VThigh from the shuttle run test was 10.5 ± 0.4 and 10.6 ± 0.4 at HRVThigh(*d* = 0.25; CI − 0.603 to 1.103), bias of the pooled data from both tests expressed as running speed was 0.02 km/h with a standard deviation of 0.3 km/h

## Results

### Literature Search

Initial search terms yielded 1206 potentially relevant articles of which 701 were screened after the removal of duplicates. Finally, 27 studies were included in the review process (Fig. 1).

### Methodological Study Quality: QUADAS2

**Fig. 2:**
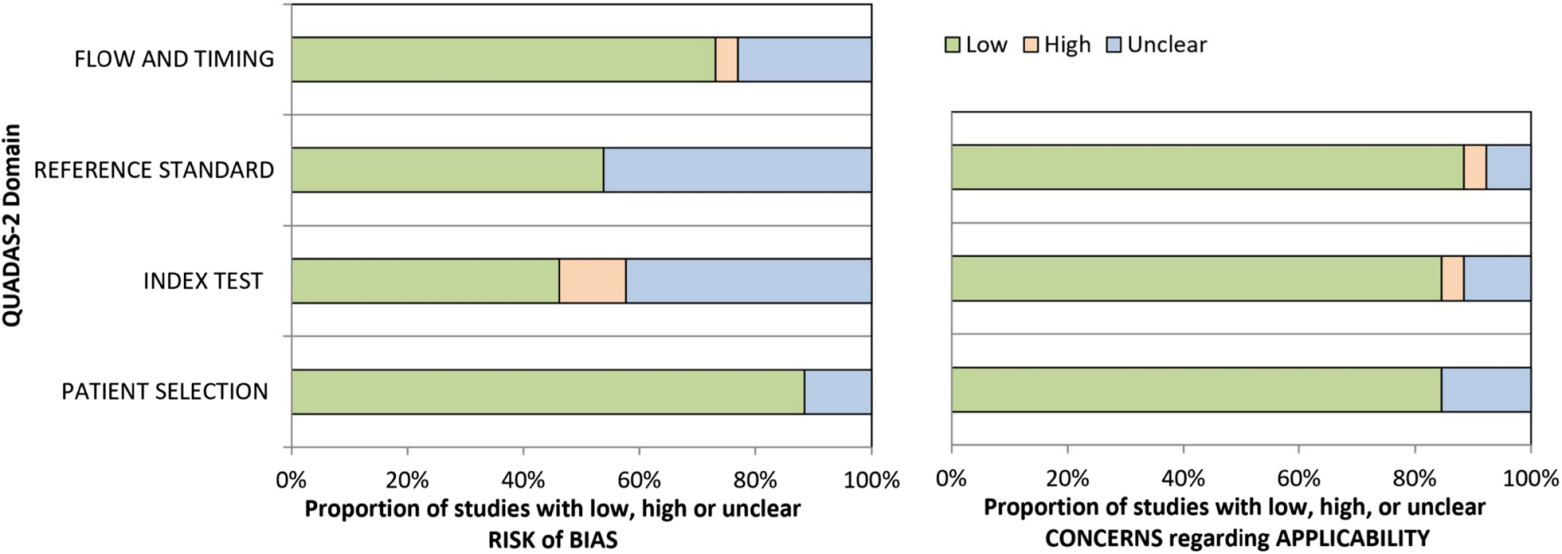
Risk of bias and applicability of included studies as assessed by QUADAS2 [68]. Relative amount of low, high and unclear risk of bias among the included studies for the domains flow and timing, reference standard, index test and patient selection

Details of the risk of bias assessment are provided in Fig. 2. Ten out of the 27 studies achieved a low risk of bias across all categories. Four studies are rated as suffering from high risk of bias in one out of the four domains and another eleven studies are rated as having “an unclear risk of bias” in two or more domains. The scoring outcomes are shown in detail in Fig. 2. All studies included in this systematic review used a within-subject design, but participant groups, reference and index tests varied considerably based upon the methodologies and parameters chosen for threshold determination.

### Methodological Study Quality: STARDHRV

The 27 studies being included in this systematic review reached an average rating of 75 ± 7% using the adapted STARD_HRV_ protocol. The highest rating achieved was 86% and the lowest 56%, while 8 studies reached ≥ 80%, 5 studies achieved ≤ 70%. While the items 1, 2, 3, 5, 7, 20 and 24 were fulfilled by all the studies, a sufficient description of the sample size determination (item 6) was only provided by one study (4%) and a stabilization period prior to the test was only addressed (item 11) in 26% of the studies. In a comparable manner, breathing rate was acknowledged (item 13) in 37% of the studies. All other items were fulfilled by 54–98% of the studies (see Additional file 1: Table S2).

### Results of Individual Studies

The result presentation in the reviewed studies was highly heterogeneous. Table 1 provides a brief overview of the included studies and their primary results. From studies that used more than one method to determine HRVT, the data of the method with the closest proximity to or best agreement with the reference method are reported both in individual results and in the synthesis, respectively.

### Results of Syntheses

In summary, 17 studies compared HRVTlow versus VTlow, 11 studies compared HRVThigh versus VThigh, six studies compared HRVTlow versus LTlow and five studies compared HRVThigh versus LThigh, respectively. Fourteen determined HRVTs based on frequency or time–frequency indices and 12 based on time-domain metrics. This includes studies that used differentapproaches/indices for HRVTlow and HRVThigh assessment. Furthermore, four studies used different nonlinear analyses for HRVT determination (Table 1).

As stated above, the result presentation in the original studies is heterogeneous, and thus, the included sample size for each part of the synthesis is reported. Weighted differences of means relative to the reference test mean of HR, power and/or speed at the lower and higher thresholds are reported in Tables 2 and 3, respectively. Further, Tables 4 and 5 show the agreement of HR, power and/or speed between VTlow and/or LTlow and HRVTlow, and between VThigh and/or LThigh and HRVThigh, respectively.

**Table 2:** The weighted relative differences (wrDiff) between heart rate (HR), power (*P*) and speed (*v*) from ‘low’ ventilatory threshold (VTlow), ‘low’ lactate threshold (LTlow) and ‘low’ heart rate variability threshold (HRVTlow); min, minimal value; max, maximal value; 90% CI, 90 percent confidence intervals; n.a., no data available; *N*, number of participants; *n*, number of studies

	HR@VTlow (%bpm)	P@VTlow (% Watt)	v@VTlow (%km/h)	HR@LTlow (%bpm)	P@LTlow (%Watt)	v@LTlow (%km/h)
HRVTlow						
wrDiff	− 0.6	0.7	0.9	0.0	1.5	− 2.7
90% CI	− 0.9 to − 0.3	0.4 to 1.0	− 0.1 to 2.0	− 3.3 to 3.3	− 3.1 to 6.0	− 4.2 to − 1.3
min	− 2.7	− 1.3	− 4.3	− 5.3	0.5	− 8.0
max	2.0	4.1	9.4	2.3	4.5	2.5
*N*/*n*	97/6	73/6	78/5	88/4	50/2	38/2

**Table 3:** The weighted relative differences (wrDiff) between heart rate (HR), power (*P*) and speed (*v*) from ‘high’ ventilatory threshold (VThigh), ‘high’ lactate threshold (LThigh) and ‘high’ heart rate variability threshold (HRVThigh); min, minimal value; max, maximal value; 90% CI, 90 percent confidence intervals; *N*, number of participants; *n*, number of studies; n.a., no data available

	HR@VThigh (%bpm)	P@VThigh (%Watt)	v@VThigh (%km/h)	HR@LThigh (%bpm)	P@LThigh (%Watt)	v@LThigh (%km/h)
HRVThigh						
wrDiff	0.3	0.6	− 0.2	2.9	0.0	5.4
90% CI	− 0.2 to 0.5	0.2 to 1.1	n.a	− 1.4 to 7.1	− 0.1 to 0.1	3.8 to 7.1
min	− 0.9	− 1.6	n.a	0	0.0	0
max	1.7	3.1	n.a	8.7	0.0	14.9
*N*/*n*	55/3	43/4	12/1	90/4	57/2	52/3

**Table 4:** The agreement between heart rate (HR), power (*P*) and speed (*v*) from ‘lower’ ventilatory threshold (VTlow), ‘lower’ lactate threshold (LTlow) and ‘lower’ heart rate variability threshold (HRVTlow) is displayed using bias with 90% confidence intervals (90% CI) and lower and upper limits of agreements (LLoA/ULoA); *N*, number of participants; *n*, number of studies; n.a., no data available

	HR@VTlow (bpm)	P@VTlow (Watt)	v@VTlow (km/h)	HR@LTlow (bpm)	P@LTlow (Watt)	v@LTlow (km/h)
HRVTlow						
Bias	1.0	1.5	0.0	2.7	3.0	0.3
90% CI	0.4–1.5	0.3–2.8	0.1–0.2	n.a	0.8–5.2	0.1–0.4
LLoA	− 10.9	− 27.6	− 1.0	− 20.4	− 27.6	− 3.2
ULoA	12.8	30.6	1.0	25.8	33.6	3.7
*N*/*n*	147/8	60/5	83/4	38/1	50/2	38/1

**Table 5:** The agreement between heart rate (HR), power (*P*) and speed (*v*) at ‘higher’ ventilatory threshold (VThigh), ‘higher’ lactate threshold (LThigh) and ‘higher’ heart rate variability threshold (HRVThigh) is displayed using bias with 90% confidence intervals (90% CI) and lower and upper limits of agreements (LLoA/ULoA); *N*, number of participants; *n*, number of studies

	HR@VThigh (bpm)	P@VThigh (Watt)	v@VThigh (km/h)	HR@LThigh (bpm)	P@LThigh (Watt)	v@LThigh (km/h)
HRVThigh						
Bias	− 4.0	1.1	− 0.0	2.5	8.0	− 0.4
90% CI	− 4.8 to − 3.1	− 0.0 to 2.2	− 0.1 to 0.0	− 0.9 to 5.8	1.1 to 14.8	− 0.5 to − 0.3
LLoA	− 17.9	− 26.8	− 0.3	− 12.1	− 27.5	− 2.1
ULoA	9.9	29.0	0.3	17.1	43.4	1.2
*N*/*n*	114/9	31/3	75/4	52/2	19/1	52/3

Concerning the comparison of VTlow and HRVTlow, which is based on pooled sample of 205 subjects from nine studies, we observed a mean correlation of *r* = 0.84 (CI 0.77–0.91) using HR in beats per minute. A correlation between HR at LTlow and HRVTlow including 59 subjects from three studies yielded *r* = 0.92 (CI 0.86–0.97). Using power in Watts for LTlow and HRVTlow including 31 subjects from two studies, the correlation coefficient was *r* = 0.87 (CI 0.86–0.87). With respect to VThigh compared to HRVThigh, a mean correlation of *r* = 0.79 (CI 0.76–0.83) was determined (i.e., based on a pooled sample of 93 subjects from four studies). Due to a lack of data in the original studies, no further pooled correlations could be determined; data from original studies can be found in Table 1.

## Discussion

This systematic review aims to provide an overview on HRVTs and their interrelations and agreements with traditional concepts of ventilatory and blood lactate thresholds. Due to the large heterogeneity of the results from 27 reviewed studies, it was not possible to include all studies in every sub-analysis of our synthesis. Overall, HRVTlow and HRVThigh show a small systematic bias compared to VTlow and VThigh, respectively. However, the limits of agreement appear to be relatively wide, especially when HR, power, and speed at the higher thresholds are considered (see Tables 4, 5). The results from the few studies that included both the comparison of HRVTlow versus LTlow and HRVThigh versus LThigh show a small systematic bias for HR and power, and a moderate systematic bias for speed at the respective thresholds, but rather larger limits of agreement. Furthermore, HRVTs seem to better reflect VTs than LTs, but this likely depends on the LT methodology and the corresponding exercise protocol [4, 9] and will be discussed in more detail afterwards. All in all, the correlations and agreements of the different HRVTs with VTs and LTs are within the range of the values that have been reported for comparisons of the latter two approaches (i.e., VTs and LTs) [71–74] and in a recent review on the relative proximity of VTs and LTs to the CP/CS concept [75]. Furthermore, it should be noted that female athletes are highly underrepresented in all method comparison studies that consider HRVTs and VTs/LTs. Thus, we are not able to draw conclusions concerning female athletes which, in turn, necessitates further threshold comparison studies in this population.

### Specification of Levels of Agreement Between HRV-Derived Thresholds and Ventilatory and/or Blood Lactate Thresholds

In general, the level of agreement of different exercise intensity thresholds that were mainly based on comparisons of HR, but also power, and speed at the respective thresholds is difficult to determine because the smallest worthwhile/meaningful change of exercise intensity in the respective intensity zone that will lead to a different training stimulus and/or have a different impact on recovery duration is ambiguous [76, 77]. For example, HR is likely to drift during any exercise bout with different amplitudes depending on exercise intensity and duration [78] and acute internal load responses to exercise are not always predictive of chronic adaptations [4]. Moreover, the markers for intensity prescription that were used in the reviewed studies (mainly HR, power, and speed) are relatively susceptible to be influenced by variability arising from different sources that may interact in complex ways. In this regard, day-to-day variability of HR has been shown to decrease with increasing exercise intensity and values vary for example in a range of 3.1–4.1% at lower running speeds and lie within 1.4–2.7% at higher running speeds, respectively [79]. In addition, the day-to-day variability in running economy is 1.77% for highly trained and 2.00% for moderately trained athletes (i.e., operationalized by the coefficients of variation [CV]) [80], and the gross efficiency of trained cyclists may vary by up to 10% (CV of 7.8–9.8% depending on exercise intensity [81]), which in both cases certainly alters the workload-HR/VO_2_ relation and therefore complicates the issue of variability of the common markers used for exercise intensity prescription. Moreover, these and other factors (e.g., temperature, oxygen partial pressure) can influence the actual physiological stimulus being triggered by a given exercise intensity [82, 83]. Thus, it seems reasonable to assume that the agreement of a certain HRVT and a corresponding reference threshold should be as good as possible and lie within the range of the basic physiological variation of the chosen exercise prescriptor (HR, VO_2_, speed/power), and/or the day-to-day variability in movement economy. In addition, it needs to be taken into account that in training practice of, e.g., well-trained athletes whose ‘lower’ and ‘higher’ thresholds occur at a high percentage of HR_max_ the corresponding HR-based exercise intensity zones may only comprise 4–8% of HR_max_ (i.e., 10 bpm for HR_max_ of 200 bpm) for heavy-to-severe domain and 10–12% of HR_max_ (24–20 bpm for HR_max_ of 200 bpm) for moderate domain, respectively [17, 84]. In this case, even a moderate level of inaccuracy (e.g., 3–5% HR_max_), especially in ‘higher’ threshold determination, can/cannot induce a frequent training stimulus with a(n) (un)desirable continuous obligatory glycolytic contribution. Furthermore, a high accuracy of the ‘lower’ threshold is also desirable as endurance athletes (especially professional athletes) typically spend a very high training volume in the moderate-intensity domain [1, 12]. Considering these issues, the following sections will discuss correlations and agreement of the different HRVTs with LT and VT approaches for ‘lower’ and ‘higher’ thresholds separately as the different reference methods (VTs and LTs) by themselves may show subtle to substantial differences depending on the different methodological settings and the sport-specific context [4, 8, 75].

### HRVTlow Versus VTlow

Seventeen studies compared HRVTlow against VTlow. Of these studies, eight used time–frequency metrics, eight time-domain metrics and one study used a nonlinear approach (DFA), while the reference methods used for VTlow were five times V-slope method, eight times based on VE/VO_2_ and three times a combination of both methods and one study used VE versus power. Our synthesis of these studies shows (i) a high mean correlation between HR at HRVTlow versus VTlow (*r* = 0.83), (ii) a small difference in weighted means, and (iii) a small bias for HR, speed and power at both thresholds, while the LoA for HR was moderately wide and acceptably wide for power and speed, respectively (see Table 4). However, when LoA for power in Watts is contextualized within the ranges of reliability measures of VTlow or LTlow, these values appear to be in the same range or are even narrower [72]. In addition, when the relative differences are used to gauge the agreement between HRVTlow and VTlow (CV of 2.0–3.5%) [71, 72], the difference between HRVTlow and VTlow may be negligible for practical applications. However, power and speed at HRVTlow seem to be slightly above those values from VTlow, while HR at HRVTlow seems to be slightly lower in the weighted mean comparison with a positive mean bias of below 1 bpm. Thus, our findings suggest that HRVTlow determination is accurate enough to set up exercise intensity zones regardless of the individual performance level [39] or training specialization [85, 86]. Moreover, HRVTlow is applicable to a variety of movement patterns such as cycling, running, walking or cross-country skiing techniques [36, 40, 86, 87]. A possible limitation of this HRVT approach may arise from movements in which the upper body activity is dominant or significantly involved in propulsion as such a movement behavior may lead to a high incidence of movement artefacts [88] and a ‘competing’ growing mechanical influence of respiratory sinus arrhythmia (RSA), muscle pump activity and cardio-locomotor coupling [33, 38, 89] that may hamper the accuracy of HRVTlow detection [86]. In this context, threshold determination based on HRV time–frequency methods might be advantageous, as the locomotor component may be separated from ventilatory activity using a specified very-high-frequency band above respiratory frequency, but our literature analysis indicated that this method has yet only been used to determine HRVThigh [89, 90]. However, despite the issue that excessive upper body movements can confound the determination of HRVTlow based on time domain metrics, no relevant systematic difference between HRVTlow determination based on time-domain or time–frequency-domain metrics has been observed. In addition, the only study that utilized a nonlinear analysis of HRVT by applying a time-varying DFA algorithm with a fixed threshold at DFAa1 = 0.75 [24], reported a small bias, acceptable limits of agreement, and a high correlation of HR and relative oxygen consumption between HRVTlow and VTlow.

In summary, exercise prescription based on HRVTlow denotes exercise intensities close to those from reference methods of VTlow over a variety of methodological settings, athletic performance levels, and endurance activities.

### HRVTlow Versus LTlow

From the six studies that compared HRVTlow against LTlow four used time-domain based approaches [35, 41, 91, 92], one used a time–frequency approach [93], and one used a nonlinear methodology [56], while the reference methods used for LTlow utilized three times the first increase from baseline, as well as baseline plus 0.2 mmol/l blood lactate concentration, Dickhuth-method [94] and 2 mmol/l blood lactate concentration, once each. The agreement between running speed at HRVTlow and LTlow was assessed in two studies and showed a moderate bias but relatively wide LoA (see Table 4). Furthermore, two studies that used a SD1-based method from Poincaré Plot analysis of HRV reported high ICC values, minimal bias but very wide LoA (see Table 1), although not all participants appeared to be included in the Bland–Altman analysis of the corresponding studies [41, 92]. Moreover, agreement of power at HRVTlow and LTlow showed a minimal bias, which is further supported by a high correlation coefficient (*r* = 87) [91]. These findings are supported by minimal, small, and moderate differences in weighted relative means for HR, power and speed at HRVTlow and LTlow, respectively (see Table 3). However, LoA were moderate for power and relatively wide for HR and speed (see Table 5). A potential reason for wider LoA is the large heterogeneity in LTlow concepts that have been used in the reviewed studies. This idea is supported by the fact that these LTlow concepts are known to differ substantially from each other [4]. Therefore, HRVTlow could be a promising approach to estimate LTlow in cycling and running for many healthy participants, but it should be considered that the individual bias can be as high as − 20 to 26 bpm [35, 93]. Based on the low number of studies that used LTlow as a reference test and the large heterogeneity in LT assessment, we are not able to derive a recommendation concerning the issue which HRVTlow method shows the closest proximity to LTlow. In general, it should be noted that the determination of lactate thresholds strongly depends on the specific lactate threshold concept and the selected exercise protocol [4, 9]. Thus, our results from the cross-comparison of different studies using different LT reference methods warrant a cautious interpretation. Interestingly, two studies [35, 93] that compared the outcomes of HRVTlow against both LTlow and VTlow observed high to very high correlations, but the correlations between HRVTlow and VTlow were slightly higher than those between HRVTlow and LTlow. This observation might be related to the direct physiological connection between breathing mechanics, respiratory sinus arrhythmia (RSA), and HRVTlow [36, 39], while the connection with LTlow is moderated by the mechanism of isocapnic buffering [95] and is therefore less straightforward. The comparison of HRVTlow and VTlow as well as HRVTlow and LTlow were comparable in terms of bias and LoA (see Table 4). Further, in both studies [35, 93] the interrelations between HRVTlow and LTlow, HRVTlow and VTlow as well as the corresponding LoAs are in a comparable range to those between LTlow and VTlow [72].

Taken together, our findings suggest that HRVTlow has a promising potential to yield a prescription of exercise intensity that is comparable to those obtained from LTlow. However, the large individual differences that may occur because of the plethora of LTlow concepts that have been used as reference methods impedes a more robust and nuanced conclusion. The above-mentioned methodological issues and the fact that women were highly underrepresented in the reviewed method comparison studies necessitates further empirically research to substantiate the evidence concerning the proximity of HRVTlow and LTlow.

### HRVThigh Versus VThigh

Our systematic review identified and included 11 studies that validated HRVThigh against VThigh. Of these studies, nine studies used time–frequency metrics [36, 39, 40, 85, 86, 89, 90, 96–98], one study used a time-domain metric [42] and one study utilized a nonlinear approach (DFA, [51]). To comapre HRVThigh to VThigh, VE/VCO_2_ was applied seven times, and two times by combining three methods, one time using VCO_2_ vs Workload and respiratory exchange ratio and one time using a web service (http://oxynetresearch.promfacility.eu) [51, 73, 99, 100]. The exercise modes in these studies include running and cycling, but also ski-mountaineering [89, 96], and cross-country skiing [86]. Furthermore, in one study a continuous 20 m shuttle-run protocol [101] was used in addition to a standard graded exercise test [98]. In general, agreement between HRVThigh and VThigh can be rated as good with moderate bias in HR, and a minimal systematic bias in power and speed. In addition, minimal differences in relative weighted means were observed. LoA were acceptable for HR and power, and small for speed (see Table 5). Only HR appears to be slightly lower at HRVThigh as compared to VThigh. The majority of studies used time–frequency methods to derive HRVThigh by determining the product of the pHF and HF plotted against exercise time. This index emphasizes the re-rise in HRV-outcome when VThigh, as operationalized by the second ventilatory threshold (VT2), is surpassed [36, 89]. Despite the high agreement between time–frequency based HRVThigh determination and VThigh in cycling and running, this method seems to be susceptible to artefacts arising from upper body movements. We noticed that, especially when the upper body was substantially involved in propulsion (e.g., cross-country skiing), the agreement between HRVThigh and VThigh was either notably lower than in other studies [86] or the detection of HRVThigh was even not possible in a considerable number of participants [96]. As mentioned earlier regarding the comparison of HRVTlow with VTlow, there is some evidence in the literature suggesting that in this specific application case advanced time-varying spectral analysis approaches (e.g., STFT, SPWVD) need to be considered to separate locomotor and respiratory frequency within the HF-band and a an extension of the HF-band of up to 2 Hz is required to be able to properly detect HRVThigh [89]. In addition to the time–frequency assessment of HRVThigh, one study [42] used a re-increase in RMSSD after the plateau that occurs around HRVTlow as a proxy for HRVThigh [42]. This time-domain based method reported a distinguishable HRVThigh for all participants under normoxic conditions and a high agreement between power at HRVThigh and VThigh, but relatively wide LoA (see Table 5. Despite these promising results, the small sample size, and the low signal-to-noise ratio in linear HRV-indices at high exercise intensities should be further evaluated to draw more robust conclusions for a practical implementation. Finally, a novel approach using nonlinear HRV-analysis in a recent study compared a fixed threshold value of DFAa1 = 0.5 as a proxy of HRVThigh with VThigh [51]. The results showed a slight systematic underestimation of HR at HRVThigh compared to VThigh (− 4 bpm) and relatively wide LoA (− 24 to 16 bpm), and a high correlation (*r* = 0.78) between HR at HRVThigh and VThigh.

Taken together, our results suggest that HRVThigh can be determined by three different approaches that yield mean values in HR, power, and speed that are comparable to and highly correlated with those obtained from VThigh approaches. However, agreement with the values obtained from VThigh is rather moderate with a more substantial individual bias as compared to the reviewed studies on method comparison studies for the ‘lower’ threshold. In addition, the methodological setting to determine heavy to severe exercise intensity seems to be more challenging for HRVT assessment, especially when upper body movement is involved [89]. Most importantly, one need to consider that recent reviews recommend the CP/CS concept as promising approach for the demarcation of the heavy to severe exercise boundary [4, 8], and that VThigh assessed by VT2 or RCP most likely overestimates CP by 6–21% [75]. As to date no study directly compared HRVThigh with CP/CS, the existing evidence derived from the reviewed studies is, in our opinion, insufficient to draw a robust conclusion on whether HRVThigh can be used to accurately separate the heavy from the severe intensity domain. Cognizant of this gap in the literature, we recommend that future method comparison studies should also incorporate CP/CS as a reference method to broaden our knowledge in this direction.

### HRVThigh Versus LThigh

Only five studies compared HRVThigh against LThigh and used a large variety of methodological approaches [41, 54, 56, 90, 92] which limits the generalizability of our conclusions. Of these studies, one used a time–frequency approach [41, 54, 56, 90, 92], two used Poincaré Plot analysis [41, 92] with direct relation to time-domain metrics [102] and two studies used a nonlinear approach [51, 56], while the reference methods used for LThigh were two times LTlow plus 1.5 mmol/l blood lactate, and once each baseline lactate plus 2 mmol/l, a fixed blood lactate value of 3.5 mmol/l and the turn point of the blood lactate curve. The threshold values that could be obtained from HRVThigh in comparison to LThigh show a small to moderate positive mean bias and differences in weighted means for HR and power (see Table 5). With regard to speed, one study used an incremental swimming test to determine time–frequency based HRVThigh by the calculation of HF power from an extended HF-band (0.4–2.0 Hz), while the mechanical influences of stroke rate and RSA were separated [90]. In comparison to LThigh, the mean difference between HR and speed at HRVThigh was small, while LoA were small to moderate (see Table 5). None of the other studies used a (time-) frequency domain-based approach for HRVThigh in comparison with LThigh and none of the other studies included female participants.

In addition, two studies conducted by the same research group used two different SD2 based methods—extracted from the corresponding Poincaré Plot—to determine HRVThigh [41, 92]. These authors then applied the Dmax-method to the SD2 curve, which is known from lactate threshold determination [103]. In comparison to LThigh the mean difference in running speed for HRVThigh was slightly above 1 km/h, and revealed substantially lower values of HR at HRVThigh (see Table 1) as well as relatively wide LoA for both running speed and HR [92]. Finally, two studies used approaches based on nonlinear HRV-analysis, namely compression entropy (H_C_) and DFAa1. Within the entropy approach [54], HRVThigh was determined as the minimum of a third order polynomial fit applied to H_C_ time series, and power at HRVThigh compared to power at LThigh (Dickhuth-method [94]) showed a small bias, rather wide LoA, and a high correlation (see Table 1). A similar pattern was reported in a study using linear regression analysis of time-varying DFAa1 and a threshold value of 0.5 to determine HRVThigh [56]. In addition, the systematic bias between HR at HRVThigh and LThigh was small and correlation was high, whereas the LoA were rather wide ([56], see also Table 1). The above-presented findings suggest that in some individuals both thresholds did not accurately match. However, it remains unclear whether this difference is an issue of the LThigh or HRVThigh concept in general or whether the chosen exercise protocol plays the particularly decisive role. Principally, the specific concept of LThigh determination and the exercise protocol play an important role and are known to strongly influence the level of agreement between parameters derived from these threshold concepts [4, 8, 9]. In this context, even wider LoA were found when comparing results from VThigh and several different LThigh determination methods [72]. Furthermore, the complex interaction of various methodologies in nonlinear HRV analysis with different recording devices and artefact correction methods [43, 94, 104] might influence the accuracy of the threshold determination. Based on the above-mentioned issues and gaps in the literature, HRVThigh concepts utilizing a nonlinear analysis of HRV need a further evaluation to ensure that they can be accurately and reliably applied in a larger number of individuals, including female participants.

In summary, HRVThigh that is determined by different linear and nonlinear HRV-metrics show heterogeneous results and moderate agreement in comparison with LThigh. Compared to the other threshold comparisons being conducted in this systematic review, HRVThigh and LThigh showed the lowest level of agreement. In this regard, our systematic analysis of the literature suggests that especially the specific LThigh concept being used in comparison with HRVThigh can strongly influence the level of agreement. Additionally, in line with the comparison of HRVThigh and VThigh, it needs to be considered that no study directly compared HRVThigh with the promising reference standard of the CP/CS concept as mentioned above [4, 8]. Taking into account that LThigh compared to MLSS is likely biased by 0.5–8% at least for running exercise [74] and MLSS, in turn, is likely to underestimate CP by 11% [75], it needs to be pointed out that the existing evidence does not allow for robust conclusions on whether HRVThigh can be used to demarcate the boundary of the heavy and severe intensity domains. As proposed for the comparison of HRVThigh and VThigh, future efforts and method comparison studies that (i) incorporate CP/CS as a reference method and (ii) include female participants being currently highly underrepresented in the existing studies on the ‘higher’ threshold comparisons, are urgently needed for more reliable and nuanced conclusions on the applicability of HRVThigh.

### Practical Applications

Based on the findings of our systematic review, the utilization of HRVTs as an alternative to VTs and LTs within the exercise intensity distribution model of endurance training is ambiguous. Our review supports the notion that HRVTlow is a promising approach for healthy, male adults participating in endurance-type activities for the determination of a ‘lower’ threshold comparable to VTlow that can be used to demarcate the boundary between moderate and heavy exercise intensity. Whether HRVThigh also constitutes a promising approach to denote the boundary between the heavy and severe exercise domain, needs further evaluation by direct method comparison studies using the CP/CS approach as a reference method.

With regard to a practical application, in a best-case scenario a trained male endurance athlete with a HR_max_ of 200 bpm will train almost exactly in the same intensity zone as when the exercise intensity would have been determined using VT approaches. Theoretically, the boundary would be about 0.5% of HR_max_ higher at the lower threshold and around 1.5% higher of HR_max_ at the higher threshold. Such small differences are negligible for practical application [72]. Nevertheless, in a worst-case scenario the boundary between moderate and heavy exercise can be over- or underestimated by 6% of HR_max_, and thus, the boundary between heavy and severe exercise would be off by about 6.8% of HR_max_. Consequently, the athlete would at least partly train in the wrong intensity zone when the aim is to train at a moderate intensity but would train entirely in the wrong zone when the aim is to train at severe exercise intensity. From a theoretical point of view, such inaccuracies would even be exacerbated when a five-intensity zone training model is applied. Thus, the applied intensity will, depending on the scenario and used training intensity model, considerably over- or undershoot the desired training load and thus probably not provoke the acute physiological responses aimed for according to the targeted exercise intensity zones [1, 9].

Finally, our systematic review does not support the use of HRVTs as proxies for LTlow and LThigh, respectively. This observation is mainly related to the plethora of methodologies that have been used in both HRVT and LT assessment. Thus, future high-quality studies with larger and more diverse populations (i.e., including female participants) and more standardized methodological procedures including the use of CP/CS concept as a reference method [4, 8] are necessary to broaden our knowledge in this direction and to allow for more robust and nuanced conclusions. Considering the findings of our systematic review on HRVT methods, we recommend the application of sophisticated time–frequency methods considering both RSA and locomotor frequency, especially for the comparison with ‘higher’ thresholds from ventilatory and/or blood lactate measurements when the upper body is involved in propulsion [89, 90]. However, our summary of the current state of the literature does not allow determination of a specific exercise protocol that suits best for a specific HRVT. Consequently, further efforts are necessary to establish generally accepted exercise protocol(s) that can be used to determine specific HRVTs. Whether HRVT-based metrics represent a useful instrument that can be utilized for a real-time prescription of exercise intensity [25] or to conduct a remote performance testing (e.g., in tele-healthcare settings), and whether this holds true for female participants or different groups of patients, is a promising area of research for future high-quality studies.

### Limitations

Based on the risk of bias assessment, the overall methodological quality of the included studies appears to be limited, which, in turn, necessitates the need for more high-quality research on HRVT to draw reliable conclusions. In general, it should be kept in mind that research involving physical exercises, to a certain degree, suffers from a selection bias as only volunteers are tested, and thus, individuals being not motivated to participate in physical exercise studies are likely to be underrepresented in the samples. Furthermore, a major limitation of the reviewed studies is that female participants are underrepresented. Thus future studies are strongly encouraged to include them to evaluate whether our findings are generalizable to females. Based on the variety of HRV-threshold concepts, further research including larger cohorts and comparing different threshold approaches is warranted. Additionally, as there is no extensive research on test–retest reliabilities of the different HRVT-methods, future studies should seek to address this gap to facilitate the application of HRVT in different settings. Similarly, stability of the HRV indices used in the original investigations and their interaction with corresponding exercise protocols and types of physical exercise need to be further evaluated. Finally, HRVTs were only validated against other threshold concepts of VT and/or LT but data on the direct relation with CP/CS are lacking. In addition, data on the HRVT-based exercise prescription during constant load exercise or regarding the longitudinal effectiveness of HRVT-based intensity zones are mandatory to evaluate whether acute HRV responses to exercise are predictive of chronic adaptations. Cognizant of the above-presented limitations and gaps in the literature, the following recommendations for future investigations are given.

### Recommendations for Future Research

- We advise upcoming studies to consider a high methodological standardization and to take all aspects of the STARD_HRV_ tool [69] into account. In this context, especially when more sophisticated HRV approaches (e.g., time–frequency and/or nonlinear methods) are used, an entirely transparent reporting including sufficient information on data processing steps should be conducted.
- Except for HRVTlow VTlow, female participants were highly underrepresented in the studies included in this systematic review. Therefore, future method comparison studies should aim to evaluate HRVT determination in females.
- Based on the limited number of available high-quality studies and being aware of their limitations, further research should investigate the influence of different exercise protocols and types of physical exercise on HRVTs. Additionally, more research on test–retest-reliability of HRVTs is needed.
- Concerning the validity of HRVTs for exercise prescription, future studies should seek to elucidate whether VT and/or LT concepts represent an adequate reference standard for a method comparison study with HRVTs, especially when the ‘higher’ threshold to partition the heavy from the severe exercise intensity domain is addressed [4, 8]. In this regard, it should also be noted that threshold concepts use transition points from specific physiological subsystems (e.g., metabolism, ventilation), and therefore related thresholds derived from different subsystems might not necessarily need to show a close agreement [105]. On the basis of “network physiology” approaches [106] a gold standard internal load metric for organismic system demands as a more comparable construct for ANS-derived thresholds like HRVT is still missing. Comparable to recent comparisons of ventilatory- and HR-based training studies [107, 108], we recommend that future high-quality longitudinal studies aim to elucidate the effectiveness of HRVTs for training prescription by comparing them with established LT-, VT- and/or CP/CS-based training prescription.

## Conclusion

In summary, the findings of our systematic review and data synthesis reveal that HRVTs for healthy, male adults participating in endurance-type activities show a general high overall correlation but very heterogeneous degree of agreement with established VTs and/or LTs. The agreement strongly varies with the chosen reference method and/or HRVT approach. Due to the large overall heterogeneity in methodological quality and the rather small sample sizes in the reviewed method comparison studies, HRVT cannot be used interchangeably with traditional threshold concepts of VT and/or LT. However, based on the evidence of the current systematic review, it seems reasonable to conclude that HRVTlow is a promising approach for the determination of a ‘lower’ threshold comparable to VTlow that could be used to demarcate the boundary between moderate and heavy exercise intensity. Considering only VT approaches, HRVThigh is also able to denote the boundary between the heavy and severe exercise domain, although this assumption needs further empirical evaluation. Based on the plethora of methodological approaches that have been used to establish LT and HRVT, the use of HRVTs as proxies for LTlow and LThigh cannot be supported yet. In particular, the lack of evaluation by direct method comparison studies with the CP/CS approach impedes the evaluation of HRVT methods for ‘higher’ threshold determination and therefore reliable conclusions on its usefulness to delineate the heavy from the severe intensity domain. Thus, to further substantiate the available evidence on HRVTs and to allow for a generalization of our findings (e.g., to females), future rigorously standardized high-quality trials with larger samples, more diverse subjects and more standardized reference approaches are required.

## Supplementary Information

**Additional file 1**: Item description of the modified Standard for Reporting Diagnostic Accuracy Studies Guidelines for Heart Rate Variability Research (STARDHRV) by Dobbs et al. [69] based on Cohen et al. [70].

## Data Availability

Data are available from the corresponding author on reasonable request.

## Abbreviations

ANS: Autonomic nervous system
AR: Autoregressive modeling
CEn: Compression entropy
CP: Critical power
CS: Critical speed
CV: Coefficient of variation
DFA: Detrended fluctuation analysis
DFAa1: Short-term scaling exponent alpha 1 of detrended fluctuation analysis
FFT: Fast Fourier transformation
GET: Gas exchange threshold to indicate an excess production of CO2 (most often V-slope of VCO2/VO2) as a boundary between moderate and heavy intensity exercise [100]
HF: High-frequency power
HR: Heart rate
HRV: Heart rate variability
HRVT: Heart rate variability-derived threshold for exercise intensity distribution
HRVThigh: Group of ‘higher’ heart rate variability-derived threshold as a boundary between heavy and severe intensity exercises
HRVTlow: Group of ‘lower’ heart rate variability-derived threshold as a boundary between moderate and heavy intensity exercises
LoA: Limits of agreement
LTlow: ‘Lower’ lactate threshold based on different approaches to denote onset of blood lactate concentration above baseline (e.g., LT, LT1 (baseline + 0.5, log-logLT)) as a boundary between moderate and heavy intensity exercise [4, 8]
LThigh: ‘Higher’ lactate threshold based on different approaches to denote an equilibrium of blood lactate production and elimination (e.g., IAT, LT2, MLSS), as a boundary between heavy and severe intensity exercise [4, 8]
MLSS: Maximum lactate steady state to indicate the maximum sustainable intensity that induces an equilibrium of blood lactate production and elimination (< 1 mmol/l increase in last 20 min of 30 min constant load exercise) as a boundary between heavy and severe intensity exercise [109]
OSF: Open Science Framework
pHF: Peak high frequency
PICOS: PICOS-principle (“PICOS” stands for participants (P), intervention (I), comparisons (C), outcomes (O), and study design (S))
PRISMA: Preferred Reporting Items for Systematic Reviews and Meta-Analyses
QUADAS: Quality Assessment of Diagnostic Accuracy Studies
RCP/VT2: Respiratory compensation point/‘second’ ventilatory threshold to denote hyperventilation as a consequent to a concomitant increase in blood lactate and H+ above disposal (most often second breakpoint in VE, clear one in VE/VCO2, fall of PETCO2) as a boundary between heavy and severe intensity exercise [4, 8]
RMSSD: Root mean square of successive differences
RQA: Recurrence quantification analysis
RSA: Respiratory sinus arrhythmia
SampEn: Sample entropy
SD1: Standard deviation one of Poincaré Plot
SD2: Standard deviation two of Poincaré Plot
SPWVD: Smoothed-pseudo Wigner–Ville distribution
STARD: Standard for Reporting of Diagnostic Accuracy Studies
STFT: Short-term Fourier transform
VT/VT1: (‘First’) ventilatory threshold based on different ventilatory methods to indicate a (‘first’) nonlinear increase in minute ventilation (VE) (most often considering VE/VO2, PETO2 and PETCO2) as a boundary between moderate and heavy intensity exercise [4]
VThigh: ‘Higher’ ventilatory threshold to denote hyperventilation as a consequent to a concomitant increase in blood lactate and H+ above disposal (e.g., RCP, VT2) as a boundary between heavy and severe intensity exercise [4, 8]
VTlow: ‘Lower’ ventilatory threshold based on different ventilatory/gas exchange methods to indicate an excess production of CO2 (e.g., VT, GET (V-slope of VCO2/VO2)) as a boundary between moderate and heavy intensity exercise [4]
