# Supplementary material for: Heart Rate Variability-Derived Thresholds for Exercise Intensity Prescription in Endurance Sports: A Systematic Review of Interrelations and Agreement with Different Ventilatory and Blood Lactate Thresholds
Source: Sports Med Open. 2023 Jul 18;9:59. doi: 10.1186/s40798-023-00607-2 (PMC10354346; doi:10.1186/s40798-023-00607-2)
Supplement: Supplementary file 1 — Additional file 1: Item description of the modified Standard for Reporting Diagnostic Accuracy Studies Guidelines for Heart Rate Variability Research (STARDHRV) by Dobbs et al. [69] based on Cohen et al. [70]. [file 40798_2023_607_MOESM1_ESM.docx]

**Electronic supplement**

**Electronic Supplementary Material Table S1:** Item Description of the modified Standard for Reporting Diagnostic Accuracy Studies Guidelines for Heart Rate Variability Research **(**STARD_HRV_) by Dobbs et al. 2019 based on Cohen et al., 2016. New modifications specific to this study are indicated by bold item numbers and changes / additions are indicated in bold letters.

| **Item** | **Explanation of Quality Item and Scoring Details** | **Source** |
| --- | --- | --- |
| **Item** | **Explanation of Quality Item and Scoring Details** | **Source** |
| **1** | The research study should identify as a validation study of a novel device as referenced to “gold standard” **ventilatory or lactate thresholds**. *Worth “1” point.* | **Modified, STARD_HRV_ (Dobbs et al., 2019) item 1** |
| 2 | The abstract should be formatted to include the study objectives, design, methods, results, and conclusions in systematic order. *All must be present to receive a score of “1”.* | **STARD_HRV_ item 2** |
| 3 | *The background along with the intended use should both be described in order to receive a score of “1” point.* | **STARD_HRV_ item 3** |
| 4 | The study objectives and hypotheses should be stated. *Worth “0.5”point each, 1 point total.* | **STARD_HRV_ item 4** |
| 5 | The Study must be within-subject design. *Worth “1” point*. | **STARD_HRV_ item 5** |
| 6 | An explanation of how the intended sample size was determined should be provided (e.g. G*Power 3). *Worth* “1” point. | **STARD_HRV_ item 6** |
| 7 | Acknowledge of participant eligibility criteria or any special considerations must be provided. *Worth “1” point*. | **STARD_HRV_ item 7** |
| 8 | Pre-testing guidelines should be provided in a specific manner to allow for study replication. *Worth “1” point*. | **STARD_HRV_ item 8** |
| **9** | The setup of the reference standard and index **test** should be described in sufficient detail to allow replication. *Detailed description of both is required to receive a score of “1”. Partially or only ONE* ***test*** *being fully described results in a score of “0.5”.* | **Modified, STARD_HRV_ (Dobbs et al., 2019) item 9** |
| 10 | The time of day and environmental conditions during sampling should be described*. Worth “0.5” each, 1 points total.* | **STARD_HRV_ item 10** |
| 11 | A stabilization period (e.g. 5min) prior to sampling HRV should be addressed. Worth “1” point. | **STARD_HRV_ item 11** |
| 12 | The raw sampling rate and length of collection should be described. *Worth “0.5” point each, 1 point total.* | **STARD_HRV_ item 12** |
| 13 | Breathing rate should be acknowledged if it was controlled or not (e.g. breathing 12 beats/min or freely breathing). *Worth “1” point.* | **STARD_HRV_ item 13** |
| 14 | A description or reference of how comparison calculations were performed should be provided. *Worth “1” point.* | **STARD_HRV_ item 14** |
| 15 | An explanation of missing data, along with the % missing and how it was handled should be provided. *A total of “1” point. If partial information is provided, give a score of “0.5”.* | **STARD_HRV_ item 15** |
| 16 | A description should be provided on how the interbeat artifacts were identified. *Worth “1” point.* | **STARD_HRV_ item 16** |
| 17 | The artifact cleaning method and the percentage of beats corrected should be fully described. *Worth “0.5” points each, 1 point total.* | **STARD_HRV_ item 17** |
| 18 | A description of each metric used and how they were calculated (e.g. log transformed), along with the software used should be provided. *Each worth “0.5”point, 1 point total.* | **STARD_HRV_ item 18** |
| **19** | An explanation of which frequency bands were measured and how they were analysed should be provided (e.g. Fast Fourier Transform or Autoregressive modelling). **For non-linear indices further information on calculation settings must be provided (e.g window length and time shift in detrended-fluctuation analysis. For time domain indices this item is not applicable**. *Worth “1” point.* | **Modified, STARD_HRV_ (Dobbs et al., 2019) item 19** |
| 20 | The baseline demographics of participants (e.g. age, sex, BMI, height, weight) should be provided. *Worth “1” point.* | **STARD_HRV_ item 20** |
| **21** | The mean and standard deviation for **outcomes of reference and index test**, along with one estimates of precision (e.g. LOA, Pearson's r or ICC) should be reported. *Worth “1” point. Provide partial points “0.5” if only one was provided.* | **Modified, STARD_HRV_ (Dobbs et al., 2019) item 21** |
| 22 | Study limitations. *Worth “1” point.* | **STARD_HRV_ item 22** |
| 23 | Implications for practice. *Worth “1” point.* | **STARD_HRV_ item 23** |
| 24 | Access to full study protocol. *Worth “1” point.* | **STARD_HRV_ item 24** |
| 25 | Sources of funding. *Worth “1” point.* | **STARD_HRV_ item 25** |

The source of the STARD_HRV_ quality item is provided, and if applicable, describes whether the item was used in its original form or modified by authors.

Abbreviations: BMI, body mass index; ICC, intra-class correlation; LoA, limits of agreement; STARD_HRV_, HRV specific version by Dobbs et al., 2019 of the Standard for Reporting Diagnostic Accuracy Studies Guidelines

| **Electronic Supplementary Material Table S2.** Item-by-item summary of methodological study quality for the included studies (n=27) using a slightly modified version of the Standard for Reporting Diagnostic Accuracy Studies modified for the use of heart rate variability (STARD_HRV_). | | | | | | | | | | | | | | | | | | | | | | | | | | | |  |
| --- | --- | --- | --- | --- | --- | --- | --- | --- | --- | --- | --- | --- | --- | --- | --- | --- | --- | --- | --- | --- | --- | --- | --- | --- | --- | --- | --- | --- |
| Study | 1 | 2 | 3 | 4 | 5 | 6 | 7 | 8 | 9 | 10 | 11 | 12 | 13 | 14 | 15 | 16 | 17 | 18 | 19 | 20 | 21 | 22 | 23 | | 24 | 25 | **Rating** | |
| Anosov et al. (2000) [34] | 1 | 1 | 1 | ½ | 1 | 0 | 1 | 0 | 1 | 0 | 0 | 1 | 1 | 0 | 1 | 1 | 1 | 1 | 1 | 1 | 0 | 1 | 0 | | 1 | 1 | **70%** | |
| Blain et al. (2005) [39] | 1 | 1 | 1 | ½ | 1 | 0 | 1 | 1 | 1 | ½ | 0 | 1 | 1 | 1 | 1 | 1 | ½ | 1 | 1 | 1 | 0 | 1 | 1 | | 1 | 1 | **82%** | |
| Cassirame et al. (2015) [96] | 1 | 1 | 1 | 1 | 1 | 0 | 1 | 0 | 1 | ½ | 0 | 0 | 1 | 1 | 1 | 1 | ½ | 1 | 1 | 1 | 1 | 1 | 0 | | 1 | 1 | **76%** | |
| Cottin et al. (2007) [40] | 1 | 1 | 1 | 1 | 1 | 0 | 1 | 0 | 1 | 0 | 0 | 1 | 1 | 1 | 1 | 1 | ½ | 1 | 1 | 1 | 1 | 1 | 0 | | 1 | 1 | **78%** | |
| Cottin et al. (2006) [36] | 1 | 1 | 1 | 1 | 1 | 0 | 1 | 1 | 1 | 1 | 0 | 1 | 1 | 1 | 1 | 1 | ½ | 1 | 1 | 1 | 1 | 0 | 0 | | 1 | 0 | **78%** | |
| Cunha et al. (2014) [87] | 1 | 1 | 1 | 1 | 1 | 0 | 1 | 1 | 1 | 1 | 1 | 1 | 0 | 1 | 0 | 1 | 1 | 1 | n/a | 1 | 1 | 1 | 0 | | 1 | 1 | **83%** | |
| Di Michele et al. (2012) [90] | 1 | 1 | 1 | ½ | 1 | 0 | 1 | 1 | 1 | 1 | 0 | 1 | 1 | 1 | 1 | 1 | 1 | 1 | 1 | 1 | 1 | 1 | 1 | | 1 | 0 | **86%** | |
| Dourado et al. (2010) [110] | 1 | 1 | 1 | ½ | 1 | 0 | 1 | 0 | 1 | 0 | 0 | 1 | 0 | 1 | 0 | 1 | 1 | 1 | n/a | 1 | 1 | 1 | 1 | | 1 | 1 | **73%** | |
| Dourado & Guerra (2013) [111] | 1 | 1 | 1 | ½ | 1 | 0 | 1 | 1 | 1 | ½ | 1 | ½ | 0 | 1 | 1 | 1 | ½ | 1 | n/a | 1 | 0 | 1 | 1 | | 1 | 1 | **76%** | |
| Garcia-Manso et al. (2008) [97] | 1 | 1 | 1 | ½ | 1 | 0 | 1 | 1 | 1 | ½ | 0 | ½ | 1 | 1 | 0 | 0 | 0 | 1 | 1 | 1 | 0 | 0 | 1 | | 1 | 0 | **62%** | |
| Garcia-Tabar et al. (2013) [91] | 1 | 1 | 1 | ½ | 1 | 0 | 1 | 1 | 1 | 1 | 0 | ½ | 0 | 1 | 0 | 1 | ½ | 1 | n/a | 1 | 1 | 1 | 1 | | 1 | 0 | **72%** | |
| Granell & De Vito (2018) [112] | 1 | 1 | 1 | ½ | 1 | 0 | 1 | 1 | 1 | 1 | 0 | 1 | 0 | 1 | 1 | 1 | 1 | 1 | 1 | 1 | 0 | 1 | 1 | | 1 | 1 | **82%** | |
| Hamdan et al. (2016) [54] | 1 | 1 | 1 | ½ | 1 | 0 | 1 | 0 | 1 | ½ | 0 | 0 | 0 | 1 | 0 | 0 | 0 | 1 | 1 | 1 | 1 | 0 | 0 | | 1 | 1 | **56%** | |
| Karapetian et al. (2008) [35] | 1 | 1 | 1 | ½ | 1 | 0 | 1 | 1 | 1 | 1 | 0 | 1 | 0 | 1 | 1 | 1 | 1 | 1 | 1 | 1 | 0 | 1 | 1 | | 1 | 0 | **78%** | |
| Mankowski et al. (2016) [42] | 1 | 1 | 1 | 1 | 1 | 0 | 1 | 1 | 1 | 1 | 1 | ½ | 0 | 1 | 0 | 0 | 0 | 1 | n/a | 1 | 0 | 1 | 1 | | 1 | 0 | **69%** | |
| Mateo-March et al. (2022) [56] | 1 | 1 | 1 | ½ | 1 | 0 | 1 | 0 | 1 | 0 | 1 | 1 | 0 | 1 | 1 | 1 | 1 | 1 | 1 | 1 | 1 | 1 | 1 | | 1 | 1 | **76%** | |
| Mendia-Iztueta et al. (2016) [86] | 1 | 1 | 1 | ½ | 1 | 0 | 1 | 0 | 1 | 0 | 0 | 1 | 1 | 1 | 1 | 1 | 0 | ½ | 1 | 1 | 0 | 0 | 1 | | 1 | 1 | **68%** | |
| Mourot et al. (2014) [89] | 1 | 1 | 1 | 1 | 1 | 0 | 1 | 0 | 1 | 0 | 0 | ½ | 1 | 1 | 1 | 1 | ½ | 1 | 1 | 1 | 1 | 1 | 1 | | 1 | 1 | **80%** | |
| Nascimento et al. (2017) [41] | 1 | 1 | 1 | 1 | 1 | 0 | 1 | 1 | 1 | 1 | 1 | ½ | 0 | 1 | 0 | 1 | ½ | 1 | n/a | 1 | 1 | 1 | 1 | | 1 | 0 | **79%** | |
| Nascimento et al. (2019) [92] | 1 | 1 | 1 | 1 | 1 | 0 | 1 | 1 | 1 | 1 | 0 | ½ | 0 | 1 | 0 | 0 | 0 | 0 | n/a | 1 | 1 | 1 | 1 | | 1 | 1 | **69%** | |
| Park et al. (2014) [113] | 1 | 1 | 1 | ½ | 1 | 1 | 1 | 1 | 1 | ½ | 0 | ½ | 0 | 1 | 0 | 0 | 0 | 1 | 0 | 1 | 0 | 1 | 1 | | 1 | 0 | **62%** | |
| Queiroz et al. (2017) [115] | 1 | 1 | 1 | 1 | 1 | 0 | 1 | 1 | 1 | 1 | 1 | ½ | 0 | 1 | 0 | 1 | ½ | 1 | n/a | 1 | 1 | 1 | 0 | | 1 | 1 | **79%** | |
| Ramos-Campo et al. (2018) [85] | 1 | 1 | 1 | ½ | 1 | 0 | 1 | 1 | 1 | 1 | 0 | 1 | 0 | 1 | 1 | 1 | ½ | 1 | 1 | 1 | 1 | 1 | 1 | | 1 | 0 | **80%** | |
| Rogers et al (2021) [24] | 1 | 1 | 1 | ½ | 1 | 0 | 1 | 1 | 1 | 1 | 0 | 1 | 0 | 1 | 1 | 1 | 1 | 1 | 1 | 1 | 1 | 1 | 1 | | 1 | 0 | **82%** | |
| Rogers et al. (2021) [51] | 1 | 1 | 1 | ½ | 1 | 0 | 1 | 1 | ½ | 1 | 0 | 1 | 0 | 1 | 1 | 1 | 1 | 1 | 1 | 1 | 1 | 1 | 0 | | 1 | 1 | **80%** | |
| Shiriashi et al. (2018) [93] | 1 | 1 | 1 | ½ | 1 | 0 | 1 | 1 | 1 | 1 | 1 | 1 | 0 | 1 | 0 | 0 | 0 | 0 | 0 | 1 | 1 | 1 | 1 | | 1 | 1 | **70%** | |
| Stergiopoulos et al. (2021) [98] | 1 | 1 | 1 | 1 | 1 | 0 | 1 | 1 | 1 | 0 | 0 | 1 | 1 | 1 | 1 | 1 | ½ | 1 | 1 | 1 | 1 | 0 | 1 | | 1 | 0 | **78%** | |
| Total percent per question | 100 | 100 | 100 | 69 | 100 | 4 | 100 | 70 | 98 | 63 | 26 | 76 | 37 | 96 | 59 | 78 | 54 | 91 | 89 | 100 | 67 | 81 | 70 | | 100 | 59 | **75%** | |
| n/a, not applicable due to the study design. | | | | | | | | | | | | | | | | | | | | | | | |  |  |  |  |  |
